# A household survey of the prevalence of subjective cognitive decline and mild cognitive impairment among urban community-dwelling adults aged 30 to 65

**DOI:** 10.1038/s41598-024-58150-3

**Published:** 2024-04-02

**Authors:** Kanokporn Pinyopornpanish, Nida Buawangpong, Atiwat Soontornpun, Kanittha Thaikla, Chanapat Pateekhum, Nopakoon Nantsupawat, Nutchar Wiwatkunupakarn, Wichuda Jiraporncharoen, Chaisiri Angkurawaranon

**Affiliations:** 1 Department of Family Medicine, Faculty of Medicine, Chiang Mai University, 110 Inthawarorot Rd., Sriphum, Muang, Chiang Mai, 50200 Thailand; 2 Division of Neurology, Department of Internal Medicine, Faculty of Medicine, Chiang Mai University, Chiang Mai, 50200 Thailand; 3 Research Institute for Health Sciences, Chiang Mai University, Chiang Mai, 50200 Thailand; 4 Global Health and Chronic Conditions Research Group, Chiang Mai University, Chiang Mai, 50200 Thailand

**Keywords:** Household survey, Community-dwelling adult, Cognitive impairment, Subjective cognitive decline, Neuropsychiatric symptom, Neurological disorders, Neurological manifestations

## Abstract

While it is possible to detect cognitive decline before the age of 60, and there is a report indicating that certain cognitive abilities peak in one's 30s, the evidence regarding cognitive problems in populations younger than 65 years is scarce. This study aims to (1) determine the proportion of community-dwelling adults with different cognitive status, and (2) determine the prevalence of neuropsychiatric behaviors. A population-based survey was conducted in Chiang Mai, Thailand. Individuals aged 30 to 65 were recruited and assessed for demographic data, memory complaints, cognitive performance, and neuropsychiatric symptoms using self-reported questionnaires. In a total of 539 participants, 33.95% had mild cognitive impairment (MCI), 7.05% had subjective cognitive decline (SCD), and 52.50% had neuropsychiatric symptoms. The risk of MCI increased with age, and neuropsychiatric symptoms were significantly higher in those with MCI or SCD than in those without (*p* < 0.001). The most common complaints were sleep problems, anxiety, and irritability. Screening for MCI in adults aged < 65 years might be useful. However, further investigation on the appropriate age to screen and the program’s cost-effectiveness is suggested.

## Introduction

Dementia, or major neurocognitive disorder, is a progressive neurological disorder with deterioration in cognitive function. It is one of the major causes of disability and dependency among older people globally^1^. Nearly 60% of people with dementia live in low- and middle-income countries, including Thailand. The progression of the disease has, not only physical and psychosocial impact to the people with dementia, but also on the family and caregivers. Screening for cognitive impairment might help patients and caregivers prepare for managing the symptoms and consequences of future dementia.

Before the patient develop dementia, there are two clinical stages of cognitive impairment that might occur include subjective cognitive decline (SCD) and mild cognitive impairment (MCI). SCD refers to a self-reported experience of worsening in their cognition with the absence of objective cognitive deficits^2^. Mild cognitive impairment (MCI), or the recent term is minor neurocognitive disorder, is a term describing a stage of having some degree of neurodegenerative disorder involving cognitive function but the daily function is not sufficiently disrupted to meet the criteria of dementia^3^. MCI has been proposed as an initial or prodromal stage of dementia. These two stages of clinical stage of cognitive decline are gradually gaining in interest as both of them could impact on the self-care behaviors and quality of life of people who are living with these conditions^4–7^ and could progress to dementia in later years^8,9^.

Evidence has shown that people with subjective cognitive decline and mild cognitive impairment are at increased risk of progression to dementia. The incidence rate of individuals with SCD to develop dementia is 8.59 per 1000 person-years when compared with the rate of 5.66 per 1000 person-years among subjects without SCD^10^. At the stage of MCI, there were reports that about 10–20% of the people with MCI could progress to dementia in a year few years later when compared to 1–2% in general population^11–14^.

Apart from cognitive symptoms, individual with cognitive impairment could have neuropsychiatric symptom which have been reported in those with MCI and dementia^15^. About 35–85% of patients with MCI have at least one neuropsychiatric symptom^16^. The presence of these symptoms in MCI is also the risk factors for progression to dementia^17^. These symptoms usually play role as one of the leading causes of caregiver burden^18^.

Internationally, the prevalence of MCI ranged from 0.5 to 41.8%^19^. In Asia, a recent meta-analysis and systematic review study has been undertaken to determine the prevalence of MCI among community-dwelling Chinese populations^20^. The number were ranging from 1.21 to 33.03%. More than half of the studies restricted participants aged over 60 years. A few studies in Thailand reported the prevalence which were slightly higher than the international reports. They were ranging from 16.7 to 43.5% and even higher in the Northern part which was high at around 71.4%^21^. While cognitive decline can be identified before aged 60 years^20^, and there is a report suggesting that some cognitive performance peaks in one’s 30s^22^, the evidence about MCI and SCD in population younger than 65 years is scarce. A report among congenital heart disease survivors found that 5% had MCI^23^. Knowing prevalence of these spectrum of cognitive changes and prevalence of neuropsychiatric symptoms in community dwelling adults is important to estimate the potential number of people who having increased risk of developing dementia and number of family living with individual with potential cognitive decline.

Therefore, this study aims to determine the proportion of individuals with normal cognition, subjective cognitive complaint, and mild cognitive impairment, and also to determine the prevalence of neuropsychiatric behaviors among community-dwelling adults aged between 30 and 65 years in urban area of Chiang Mai, Thailand.

## Methods

### Study design

A population-based survey was conducted in Chiang Mai province, the largest province in the Northern region of Thailand.

### Sampling and sample size calculations

A stratified two-stage cluster sampling technique was done. The enumeration areas (EAs) were districts in the city center of Chiang Mai Province (the primary sampling units). Thirty-six EAs were randomly selected using probability proportional to the size of the population in each unit. Twenty households (the secondary sampling units) for each cluster were randomly selected using systematic sampling. This allowed 720 households to be included in the study. All family members aged between 30 and 65 years were recruited.

### Data collection and measurements

Demographic and sociodemographic data (age, gender, education, underlying diseases, functional status and physiological parameters) were obtained from interviews and physical examination performed by three trained research assistants between September 2020 and December 2020. For functional status, in this household survey we used a brief question to categorize the participants into (1) total dependence (all ADLs requiring assistance), (2) partial dependence (at least 1 ADL requiring assistance but not all), and (3) independence^24^. The population with independent living and no previous diagnosis of dementia were further recruited for the analysis. In addition to general demographic data, the survey obtained information on self-reported memory complaint from participants and family member. The cognitive performance and neuropsychiatric symptoms were assessed. The questionnaires were included in the Supplementary file 1.

Objective cognitive assessment was performed by using *the Thai version of Montreal Cognitive Assessment* (MoCA) which has a high sensitivity and specificity for detecting mild cognitive impairment^25^. The battery consists of many dimensions of cognitive function including memory, attention, language, orientation, executive function, naming, and abstract thinking. In this version, the sum score of 25 and over refers to negative for testing. This MoCA-Thai version was validated in 2009^26^ and has been used in recent studies^27–30^, The tool is available online at https://mocacognition.com/paper for any languages, including Thai and English.

SCD was diagnosed by a self-report on experiencing memory decline but cognitive test was negative (MoCA score ≥ 25). MCI diagnostic criteria based on revised criteria in 2011^31^ included the following: (1) concern by participant or family member regarding cognitive change, (2) cognitive impairment in one or more domains, (3) normal activities of daily living, and (4) absence of dementia. As mentioned earlier, in this study, the cognitive performance was assessed by MoCA test. Participant who received MoCA score ≥ 25 and had no report of memory impairment were classified as normal.

Neuropsychiatric symptoms were assessed using a self-reported questionnaire adapted from the Neuropsychiatric Inventory-Questionnaire (NPI-Q) by simply asking that “In the past month, have you been feeling yourself change in any of these kinds of behaviors or moods?” The 12 domains of symptoms included delusion, hallucination, depression, anxiety, euphoria, apathy, agitation or aggression, disinhibition, irritability, repetition, sleep problem, and eating problem. Questions can be answered with “yes” or “no”. The answer “yes” to any symptom reflect that the participant had that neuropsychiatric symptom.

### Statistical analysis

The data were analyzed with STATA version 15.1. Descriptive statistics including frequency, percentage, mean and standard deviation (SD), were used to analyze demographic data. Descriptive statistics were used to describe the sampled population in terms of age, gender, education, underlying diseases, and physiological parameters. To infer back to the source population, weighted analysis were conducted to account for a clustering effect using the *survey* commands. Univariate analysis included Pearson’s chi-square, student *t* test, and relative risk (95% CI). To assess the association between the presence of at least one symptom of neuropsychiatric symptom (independent variable) and a cognitive problem combining MCI and SCD as a dependent variable, a multivariable logistic regression analysis was performed. This analysis was adjusted for covariates obtained from the univariable analysis, followed by a backward stepwise reduction, with the reduced model presented as the final result. A *p* value < 0.05 was considered as statistically significant.

### Ethical approval and consent to participate

This study came from the study of health service management for patients with hypertension in Thailand. Sub-study in Chiang Mai province was conducted to understand to consequence of hypertension in elderly such as dementia and mild cognitive impairment. The study was approved by the Faculty of Medicine, Chiang Mai University ethic committee (Research ID: 7528, IRB Number 308/2563). Research involving human research participants must have been performed in accordance with the Declaration of Helsinki. Informed consent was obtained from all participants.

## Results

The total of 539 participants were recruited in the analysis of study. Most of them were female (67.90%) and the average age was 51.49 years-old (SD 10.25). Around 64% of the population has highest education at high school or higher and about 56% has at least one underlying disease. Demographic data of the study samples was shown in Table 1.

**Table 1:** Demographic of study samples (unweighted data).

	Total N = 539	MCI N = 183	SCD N = 38	Normal N = 318	*P*-value
Female, n (%)	366 (67.90)	136 (74.32)	31 (81.58)	199 (62.58)	**0.004**
Age, mean ± SD	51.49 ± 10.25	54.90 ± 9.01	50.18 ± 8.75	49.69 ± 10.61	**< 0.001**
Highest education, n (%)					**< 0.001**
No education	31 (5.75)	5 (2.73)	0 (0)	26 (8.18)	
Primary school	161 (29.87)	79 (43.17)	15 (39.47)	67 (21.07)	
High school and higher	347 (64.38)	99 (54.10)	23 (60.53)	222 (70.75)	
Underlying disease, n (%)	303 (56.22)	105 (51.72)	30 (30.77)	94 (29.38)	**< 0.001**
Diabetes	61 (11.32)	30 (16.39)	2 (5.26)	29 (9.12)	**0.022**
Hypertension	133 (24.68)	62 (33.88)	6 (15.79)	65 (20.44)	**0.001**
Dyslipidemia	57 (10.58)	26 (14.21)	6 (15.79)	25 (7.86)	**0.047**
Cerebrovascular disease	2 (0.37)	2 (1.09)	0 (0)	0 (0)	0.142
Cardiovascular disease	4 (0.74)	1 (0.55)	0 (0)	3 (0.94)	0.758
Gastrointestinal disease	14 (2.60)	3 (1.64)	2 (5.26)	9 (2.83)	0.407
Hematologic disease	9 (1.67)	5 (2.73)	1 (2.63)	3 (0.94)	0.287
Other neurological disease	3 (0.56)	1 (0.55)	0 (0)	2 (0.63)	0.886
Physiological parameters					
SBP, mean ± SD	128.13 ± 18.67	128.22 ± 20.27	125.12 ± 19.51	128.44 ± 17.61	0.583
DBP, mean ± SD	82.41 ± 12.27	82.34 ± 12.64	81.83 ± 14.59	82.51 ± 11.79	0.945
WC, mean ± SD	86.27 ± 12.04	87.64 ± 13.77	83.39 ± 10.18	85.83 ± 11.08	0.083
BMI, mean ± SD	24.69 ± 4.56	25.02 ± 4.89	23.54 ± 3.11	24.64 ± 4.49	0.185

*BMI* body mass index; *DBP* diastolic blood pressure; *SCD* subjective cognitive decline; *SBP* systolic blood pressure; *MCI* mild cognitive impairment; *WC* waist circumference.

Significant values are in bold.

**Figure 1:**
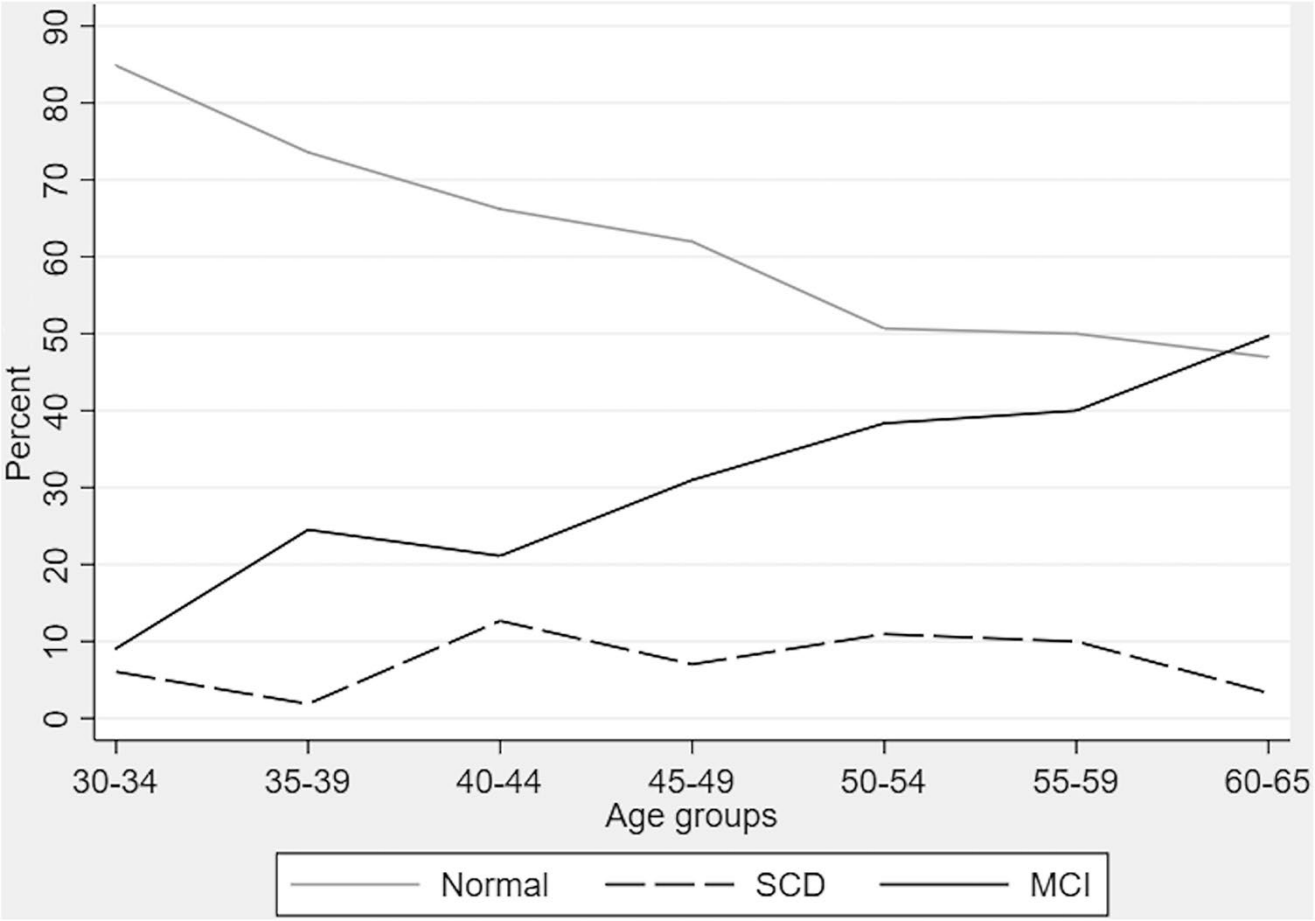
Unweighted proportion of population with normal cognition, subjective cognitive decline (SCD), and mild cognitive impairment (MCI) stratified by age; N = 539.

Figure 1 showed the proportion of adults with normal cognition, SCD, and MCI. The prevalence of MCI among adult population in our study who aged between 30 and 65 years old was 33.95% and prevalence of SCD was 7.05%. When stratified by age, the proportion of adults with MCI was increased with age (Table 2). While the prevalence was 9.57% in age group 30–34 years, it was high as 47% in age group 60–65 years. The proportion of SCD were range between 1.5 and 15.5%.

**Table 2:** Cognitive status stratified by age (weighted data).

Age group, row% (95%CI)	Total N = 539	MCI N = 183	SCD N = 38	Normal N = 318
30–34 years	n = 33	9.57 (2.89, 27.30)	4.74 (1.16, 17.35)	85.69 (68.57, 94.26)
35–39 years	n = 52	22.38 (12.67, 36.41)	1.47 (0.20, 9.84)	76.15 (62.10, 86.16)
40–44 years	n = 69	19.77 (11.68, 31.46)	14.11 (7.01, 26.34)	66.12 (53.26, 76.97)
45–49 years	n = 70	33.40 (22.58, 46.31)	8.02 (3.20, 18.68)	58.58 (45.85, 70.25)
50–54 years	n = 68	33.20 (22.66, 45.73)	15.53 (7.88, 28.34)	51.27 (38.83, 63.56)
55–59 years	n = 78	41.50 (30.48, 53.43)	11.31 (5.53, 21.77)	47.19 (35.85, 58.82)
60–65 years	n = 169	47.00 (39.15, 55.01)	4.81 (2.15, 10.42)	48.19 (40.31, 56.15)

*SCD* subjective cognitive decline; *MCI* mild cognitive impairment.

Among 183 participants with MCI and 38 participants with SCD, neuropsychiatric symptoms have been reported as high as over 60% in both groups which were significantly higher than found in population with normal cognition (*p* < 0.001). As shown in Table 3, sleep problems, anxiety, and irritability were the top three neuropsychiatric symptoms that has been reported in this population. The symptoms that were significantly different in number of self-reports between group included depression (*p* 0.001), anxiety (*p* < 0.001), apathy (*p* 0.001), agitation (*p* 0.026), irritability (*p* < 0.001), repetitive behaviors (*p* 0.003), sleep problem (*p* < 0.001), and eating change (*p* 0.015). When including MCI and SCD together, the multivariable logistic regression analysis, adjusted for age and sex, showed a significant association between neuropsychiatric symptoms and cognitive impairment as shown in Table 4.

**Table 3:** Self-report neuropsychiatric symptoms (weighted data).

Neuropsychiatric symptoms col % (95% CI)	Total N = 539	MCI N = 183	SCD N = 38	Normal N = 318	*P*-value
Have at least one symptom	n = 283	62.83^a,c^ (55.01, 70.04)	66.92^a,b^ (49.64, 80.60)	41.3^b,c^ (35.76, 47.09)	**< 0.001**
Delusion	n = 19	4.07^c^ (1.96, 8.29)	8.60 (2.67, 24.40)	1.98^c^ (0.98, 3.93)	0.067
Hallucination	n = 1	0.40 (0.06, 2.84)	0	0	0.490
Depression	n = 13	4.09^a,c^ (2.07, 7.91)	6.84^a,b^ (2.05, 20.53)	0.25^b,c^ (0.03, 1.75)	**0.001**
Anxiety	n = 50	15.9^a,c^ (11.18, 22.07)	39.42^a,b^ (24.77, 56.26)	0.75^b,c^ (0.17, 3.20)	**< 0.001**
Apathy	n = 15	4.90^a,c^ (2.66, 8.86)	3.38^a,b^ (0.83, 12.81)	0.49^b,c^ (0.12, 1.97)	**0.001**
Euphoria	n = 7	1.63 (0.49, 5.31)	0	1.76 (0.64, 4.74)	0.732
Disinhibition	n = 3	0.40 (0.06, 2.84)	1.69 (0.23, 11.25)	0.29 (0.04, 2.05)	0.340
Agitation	n = 8	1.62^a,c^ (0.60, 4.26)	5.15^a^ (1.18, 19.71)	0.49^c^ (0.12, 1.97)	**0.026**
Irritability	n = 81	27.38^a,c^ (21.15, 34.63)	39.49^a,b^ (24.71, 56.48)	4.18^b,c^ (2.37, 7.27)	**< 0.001**
Repetitive behaviors	n = 22	5.38^a,c^ (2.84, 9.62)	12.05^a,b^ (4.47, 28.65)	1.74^b,c^ (0.82, 3.61)	**0.003**
Sleep problem	n = 123	46.14^a,c^ (38.60, 53.87)	44.70^a,b^ (29.08, 61.44)	9.84^b,c^ (6.55, 14.51)	**< 0.001**
Eating change	n = 22	6.52^a,c^ (3.88, 10.75)	6.78^a,b^ (2.49, 17.13)	1.52^b,c^ (0.49, 4.59)	**0.015**

*SCD* subjective cognitive decline; *MCI* mild cognitive impairment.

*P*-value from multiple comparisons: ^a^ < 0.05 when compare MCI and SCD, ^b^ < 0.05 when compare SCD and normal, ^c^ < 0.05 when compare MCI and normal.

Significant values are in bold.

**Table 4:** The final model of multivariable logistic regression analysis assessing the association between neuropsychiatric symptoms and a cognitive problem, combining MCI and SCD.

Cognitive problem	aOR	95%CI		*P*-value
Have at least one symptom of neuropsychiatric symptom	2.59	1.79	3.76	**< 0.001**
Older age	1.05	1.03	1.07	**< 0.001**
Female	0.57	0.38	0.84	**0.005**

Significant values are in bold.

## Discussion

Our study found that MCI was also prevalent in age younger than 65 years and the prevalence was increased with age, while this phenomenon was not found in SCD. About one third of adults with SCD and MCI reported having at least one neuropsychiatric symptoms. Most neuropsychiatric symptoms reported by individual with SCD were anxiety, irritability and sleep problem whereas most individuals with MCI reported of having sleep problem, anxiety, and irritability, respectively.

The prevalence of MCI in adult population aged between 35 and 65 years was around 34%. This was high when compared to prior studies in older adults in community-dwelling population in China^20^. Even we excluded the population aged younger than 60 years, the prevalence (47%) was still higher than results from most of previous community-based researches which was usually less than 20%^32–35^. However, the prevalence is still within the upper range when compared to the review study of studies worldwide^36^. Our study employed the MoCA as the assessment tool, known for its superior ability to detect MCI compared to the MMSE. This heightened sensitivity of MoCA may have contributed to a broader range of identified cases when compared to the tools used in the most frequently referenced studies. When compare to the results from research in Thai population, the result was still lower than the prior study in Northern Thai community-dwelling older adults^21^. This could be from the diagnostic test were slightly different and they studied in older population living in rural area which could have lower education and different cognitive related lifestyle when compared to the younger population living in urban area as in our study. Therefore, they potentially have higher prevalence of MCI.

The higher prevalence of MCI could be associated with increasing age which is not surprising. This result was consistent with many studies^20,35,37^. Increasing age may increase the number of underlying disease which is associated with cognitive impairment^38^. Moreover, the factors associated with the progression of cognitive impairment through aging process includes systemic inflammation, cerebral ischemia, toxins, or neurodegeneration^39^. These leads to both molecular and structural brain change and then cognitive decline.

For SCD, our study reported that the prevalence is about 1.5–15.5% while the recent review reports the prevalence of SCD in population with old age is ranged from 10 to 88%^40^. In younger adults, aged 65 and lower, tend to have lower prevalence compared to the older age. However, in between age 30 to 65, as in our study, we could not observe this trend. Maybe in this age group, there could be some degree of cognitive change due to other causes that could occasionally occur from lifestyle and is not directly related to age change, such as, emotional disturbance or psychiatric disorder^41^, substance use^42^, or head trauma^43^.

The prevalence of neuropsychiatric symptoms were high as over 60% for both MCI and SCD. Our finding was still in line with the previous study that report the prevalence of neuropsychiatric symptoms ranging from 35 to 85% in MCI^15^. However, we could not compare the prevalence of neuropsychiatric symptoms in SCD as the evidence is still limited. It is important to assess these symptoms because they could be early symptoms/signs of dementia, especially depression, anxiety, apathy, irritability, and sleep disorder^40,44–46^. We also found that anxiety, irritability and sleep change were most reported by adults with SCD and MCI. The finding in our study could be supported by this statement. Moreover, the overall characteristic of self-reported neuropsychiatric symptoms was similar between SCD and MCI which was different from normal cognition. There might be some brain pathologies occur in these two prodromal groups but not severe enough to cause dementia. Early detection of these symptoms and provide appropriate treatment would be beneficial for patients and also allow physician to detect early dementia.

The strength of our study was that we could include a large number of participants in the study in an urban northern Thailand community-based setting. To our knowledge, this is the first study that assess the prevalence of MCI in a community-dwelling non-elderly population in Thailand. Nonetheless, the study is not without its limitations. This study used cognitive assessment tool, MoCA, which could be interfered by education level of the subject which might lead to the overestimation of the prevalence. However, MoCA is the prominent tool to detect MCI in many studies worldwide^20^ and the scoring system add up the score for those who have lower education to adjust this confounding effect. The diagnosis of MCI was not based on the most recent criteria in DSM-V, which requires physicians to exclude some symptoms that mimic MCI. However, this criterion has been widely used and cited in many research articles. In our study, neuropsychiatric symptoms were measured based on participants’ self-perception. We did not use the standard screening tool which consist of more symptoms and mostly use to interview informants, not the participants themselves which could affect the result.

In conclusion, this study reported the slightly high prevalence of SCD and MCI among middle aged adults. Even though there is no current effective treatment for MCI population, it is suggested that health care provider can start providing intervention to people with MCI believing that it could delay the cognitive decline and improve symptoms, which include neuropsychiatric symptoms^47^. Therefore, it might be useful to detect MCI in population age lower than 65 years for early detection of these cases. However, further study to find the appropriate age to start screening is still needed. The cost-effectiveness study might be useful to help design the method of screening at the community level.

### Supplementary Information

Supplementary Information.

## Data Availability

Data sets are available from the corresponding author on request due to ethical commitment.
