# Supplementary material for: A household survey of the prevalence of subjective cognitive decline and mild cognitive impairment among urban community-dwelling adults aged 30 to 65
Source: Sci Rep. 2024 Apr 2;14:7783. doi: 10.1038/s41598-024-58150-3 (PMC10987517; doi:10.1038/s41598-024-58150-3)
Supplement: Supplementary file 1 — Supplementary Information. [file 41598_2024_58150_MOESM1_ESM.docx]

**Supplementary file 1**

**Questionnaire**

1. ID________________
2. Sex ⬜ Male ⬜ Female
3. Age __________ years old
4. Nationality ___________
5. Highest education ⬜ No education ⬜ primary school ⬜ high school

⬜ Bachelor’s degree ⬜ higher

1. Underlying disease (if any, can choose more than one)

⬜ No ⬜ Diabetes ⬜ Hypertension

⬜ Dyslipidemia ⬜ Cerebrovascular disease

⬜ Cardiovascular disease ⬜ Gastrointestinal disease

⬜ Hematologic disease ⬜ Neurological disease

1. Functional status (Does the participant do these activities on their own or need any help to do these activities: feeding, bathing, grooming, dressing, bowel control, bladder control, toileting, chair transfer, ambulation and stair climbing?)

⬜ Totally dependence (cannot do any activity without help)

⬜ Partial dependence (need help on some activities)

⬜ Independence (no need help in any activity)

1. Neuropsychiatric symptoms (Is there any behavioral changes related to these symptoms in the past month? More than one answer is possible.)

⬜ No

⬜ Delusion ⬜ Hallucination ⬜ Depression ⬜ Anxiety

⬜ Euphoria ⬜ Apathy ⬜ Disinhibition ⬜ Agitation

⬜ Irritability ⬜ Repetitive behaviors ⬜ Sleep problem ⬜ Eating change

1. Complaint of memory problem ⬜ No ⬜ Yes
2. MoCA score __________
3. Physical examination
   1. SBP ____________ mmHg
   2. DBP ____________ mmHg
   3. WC ____________ cm
   4. Weight ____________ kg
   5. Height ____________ cm
